# Non-diagnostic Results of Percutaneous Transthoracic Needle Biopsy: A Meta-analysis

**DOI:** 10.1038/s41598-019-48805-x

**Published:** 2019-08-27

**Authors:** Kum Ju Chae, Hyunsook Hong, Soon Ho Yoon, Seokyung Hahn, Gong Yong Jin, Chang Min Park, Jin Mo Goo

**Affiliations:** 1 Department of Radiology, Institute of Medical Science, Research Institute of Clinical Medicine of Chonbuk National University-Biomedical Research Institute of Chonbuk National University Hospital, Jeonju, South Korea; 2 Medical Research Collaborating Center, Seoul National University Hospital, Seoul, Korea; 3 Department of Radiology, Seoul National University College of Medicine, Seoul National University Hospital, Seoul, Korea; 4 Department of Medicine, Seoul National University College of Medicine, Seoul, Korea; 5 Institute of Radiation Medicine, Seoul National University Medical Research Center, Seoul, Korea

**Keywords:** Cancer imaging, Oncology

## Abstract

Non-diagnostic results can affect the diagnostic performance of percutaneous transthoracic needle biopsy (PTNB) but have not been critically meta-analyzed yet. To meta-analyze the incidence and malignancy rate of non-diagnostic results, 3-by-2 table approaches rather than the conventional 2-by-2 approaches are needed to know its impact on the diagnostic performance of PTNB. A systematic literature search identified studies evaluating the diagnostic performance of PTNB with extractable outcomes. A total of 143 studies with 35,059 biopsies were included. The pooled incidence of non-diagnostic results was 6.8% (95% CI, 6.0–7.6%; I^2^ = 0.91). The pooled malignancy rate of non-diagnostic results was 59.3% (95% CI, 51.7–66.8%; I^2^ = 0.80), and was correlated with the prevalence of malignancy (correlation coefficient, 0.66; 95% CI, 0.42–0.91). Pooled percentage decrease of sensitivity and specificity due to non-diagnostic results were 4.5% (95% CI, 3.2–5.7%; I^2^ = 0.64) and 10.7% (95% CI, 7.7–13.7%; I^2^ = 0.70), respectively, and the pooled incidence of non-diagnostic results was 4.4% (95% CI, 3.2–5.8%; I^2^ = 0.83) in lesions ultimately diagnosed as malignancies and 10.4% (95% CI, 7.5–13.8%; I^2^ = 0.74) in benign disease. In conclusion, non-diagnostic results averagely occurred in 6.8% of PTNB and more than half of the results were malignancies. The non-diagnostic results decreased specificity and sensitivity by 10.7% and 4.5%, respectively, demanding efforts to minimize the non-diagnostic results in PTNB.

## Introduction

Percutaneous transthoracic needle biopsy (PTNB) is a safe, accurate diagnostic procedure for evaluating pulmonary lesions, with an average sensitivity of 90% and specificity of 97%^1,2^. With the introduction of advanced imaging modalities for needle guidance, the diagnostic accuracy of computed tomography (CT) fluoroscopy- and cone-beam CT-guided biopsies increased to 95.2%^3^ and 97.0%^4^, respectively.

The diagnostic accuracy of PTNB is currently assessed using a 2-by-2 table, in which the PTNB results are clearly separated into positive and negative results, and then are compared with reference standards to create the following 4 cells: true positivity, false positivity, false negativity, and true negativity^5^. However, non-contributory results can be designated as neither positive nor negative PTNB results; these occur when the PTNB specimen is non-diagnostic, meaning that it does not provide any information for differentiating malignancy from benign disease^3^. The non-contributory results are expected to affect the diagnostic accuracy but are often omitted in a 2-by-2 table approach. The simple exclusion of non-diagnostic results from the 2-by-2 table leads to an overestimation of diagnostic accuracy^6–9^.

Non-diagnostic results has not yet been critically analyzed and it can be incorporated by using a 3-by-2 table to assess diagnostic accuracy, which enables a more realistic evaluation^10^. Thus, we meta-analyzed the incidence, malignancy rate of non-diagnostic biopsy results, and its impact on the diagnostic performance of PTNB for focal lung lesions using conventional 2-by-2 and 3-by-2 table approaches to handle non-diagnostic results.

## Results

**Figure 1:**
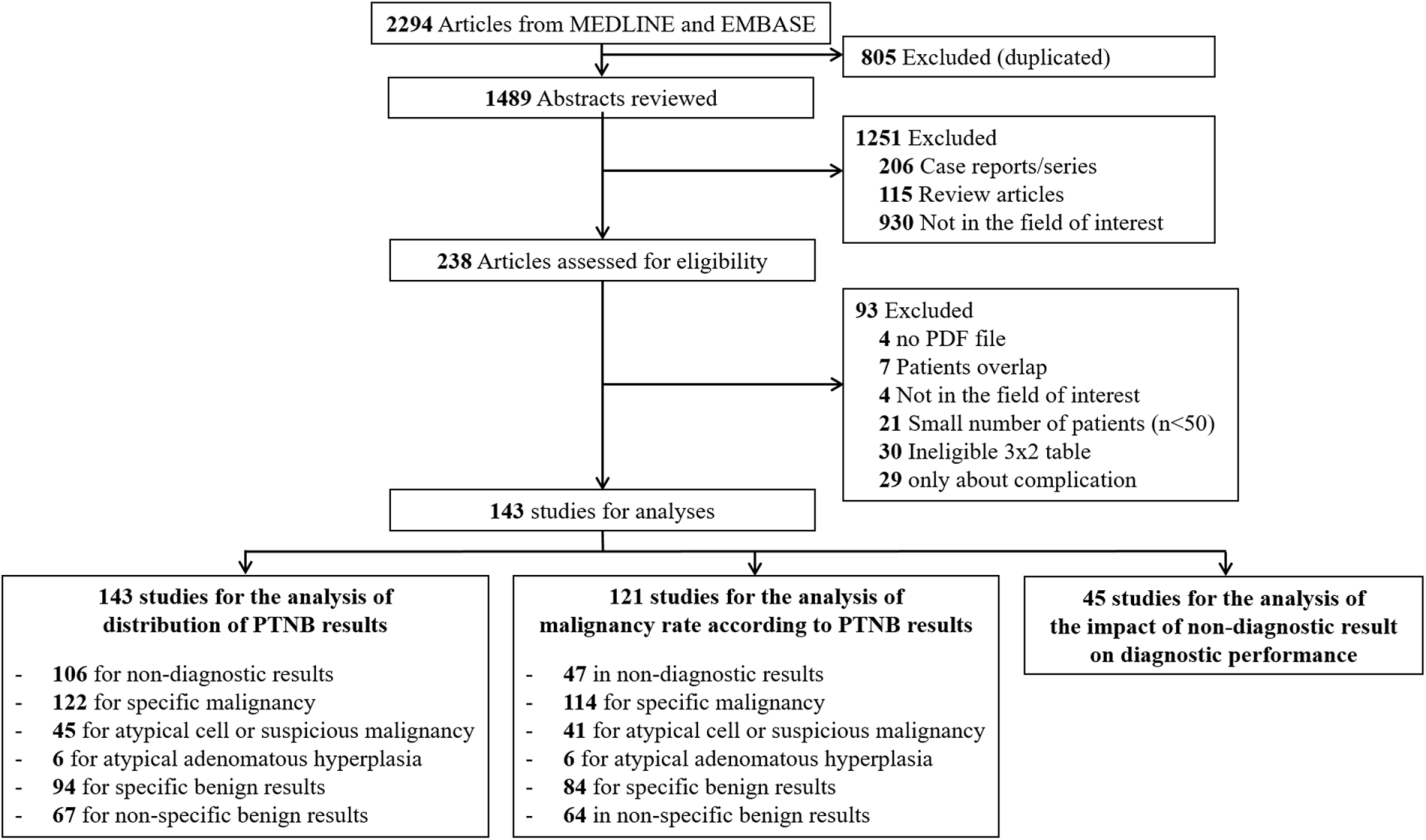
Flow diagram of the literature search.

Of the 2294 references identified in the initial database search, 143^3,4,6–9,11–147^ with 35,059 biopsies were finally included in our analysis (Fig. 1).

The baseline characteristics and results of the 143 included studies are summarized in Supplementary Table A1. The number of total attempted biopsies ranged from 50 to 994 (median, 160; interquartile range [IQR], 100–316) and the median of the median or mean size of the pulmonary lesions was 31 mm (IQR, 24–37 mm). In 84 studies, fine needle aspiration (FNA) was mainly performed, and core biopsy was primarily analyzed in 59 studies. CT was the most frequently used modality for needle guidance (n = 77), followed by fluoroscopy (n = 37), cone-beam CT (n = 12), and CT fluoroscopy (n = 5); 12 other studies used 2 or more modalities for needle guidance. The median prevalence of malignancy was 78.6% (IQR, 69.1–84.3%; range, 54.2–96.5%). When assessed by the Quality Assessment of Diagnostic Accuracy Studies (QUADAS)-2 tool, the included studies appeared to have a relatively low risk of bias in index test domain. However, the risk of bias was unclear in approximately two-fifths of included studies in patient selection, reference standard, flow and timing domains (Supplementary Fig. A1).

### Incidence of non-diagnostic and other PTNB results

The pooled incidence of non-diagnostic results was 6.8% (95% CI, 6.0–7.6%; I^2^ = 0.91; 24668 biopsies in 106 studies)^4,6–8,11,12,19,22–24,26–35,38,40–42,44–49,52,54,55,59–62,65–67,69–86,89,91–97,99–103,106–113,115–118,120,122–126,128–133,135–143,146,147^.

**Figure 2:**
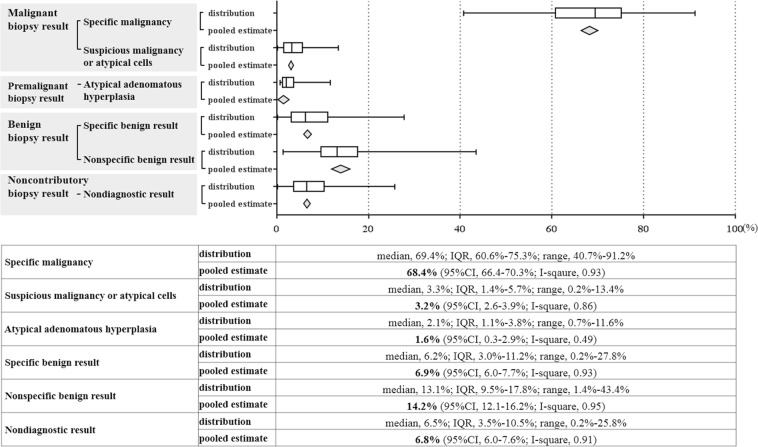
Distribution of pathology reports of biopsy specimen in percutaneous thoracic needle biopsy.

The pooled incidence rates of other pathology findings of PTNB specimens are shown in Fig. 2: specific malignancy, 68.4% (95% CI, 66.4–70.3%, I^2^ = 0.93; 29739 biopsies in 122 studies)^3,4,6–9,11–23,25–70,72–76,78,79,81–86,88–93,96,98–105,108,110,113–115,118–121,124,126–130,132–134,137–147^; atypical cells, 3.2% (95% CI, 2.6–3.9%, I^2^ = 0.86; 11032 biopsies in 45 studies)^4,9,13,14,17–20,26,27,29,30,34,37,41,42,46,47,53,64,66,69,70,74–76,78,79,85,86,89–91,98,101,104,108,115,120,121,126,129,133,144,147^; AAH, 1.6% (95% CI, 0.3–2.9%, I^2^ = 0.49; 849 biopsies in 6 studies)^8,76,84,126,128,138^; non-specific benign results, 14.2% (95% CI, 12.1–16.2%; I^2^ = 0.95; 16455 biopsies in 67 studies)^4,6,8,11,12,19,22,23,26–28,30,31,33,34,38,40,42,44,45,47–49,52,59–62,65,67,69,70,72,74–76,78,79,81–86,91,101,108,110,115,118–120,124,126,128–130,132,133,137–140,143,146–148^; specific benign results, 6.9% (95% CI, 6.0–7.7%, I^2^ = 0.93; 22390 biopsies in 94 studies)^4,6,9,12–16,18,21–23,26–28,30,31,33–40,42,43,45,46,48,50–53,56–65,67–70,72–76,79,81–83,85–88,90–93,98–101,103,104,108,110,114,115,118–121,124,126–130,132,134,139–141,143–145,147^.

On univariate meta-regression analyses, the incidence of non-diagnostic results were significantly lower with core biopsy (versus FNA; p = 0.010), CT or CTF guidance (versus fluoroscopy; p = 0.041), usage of 18 gauge needle or larger (versus 20 gauge or smaller; p = 0.137) (Supplementary Table A2). The core biopsy was significantly associated with the lower frequencies of non-specific benign results (*P* = 0.035), and higher frequencies of specific benign results (*P* = 0.001) than FNA. Lesion size significantly affected the incidence of specific malignancy, but did not affect the incidence of non-diagnostic results (Supplementary Table A2).

### Final malignancy rate of non-diagnostic and other PTNB results

**Figure 3:**
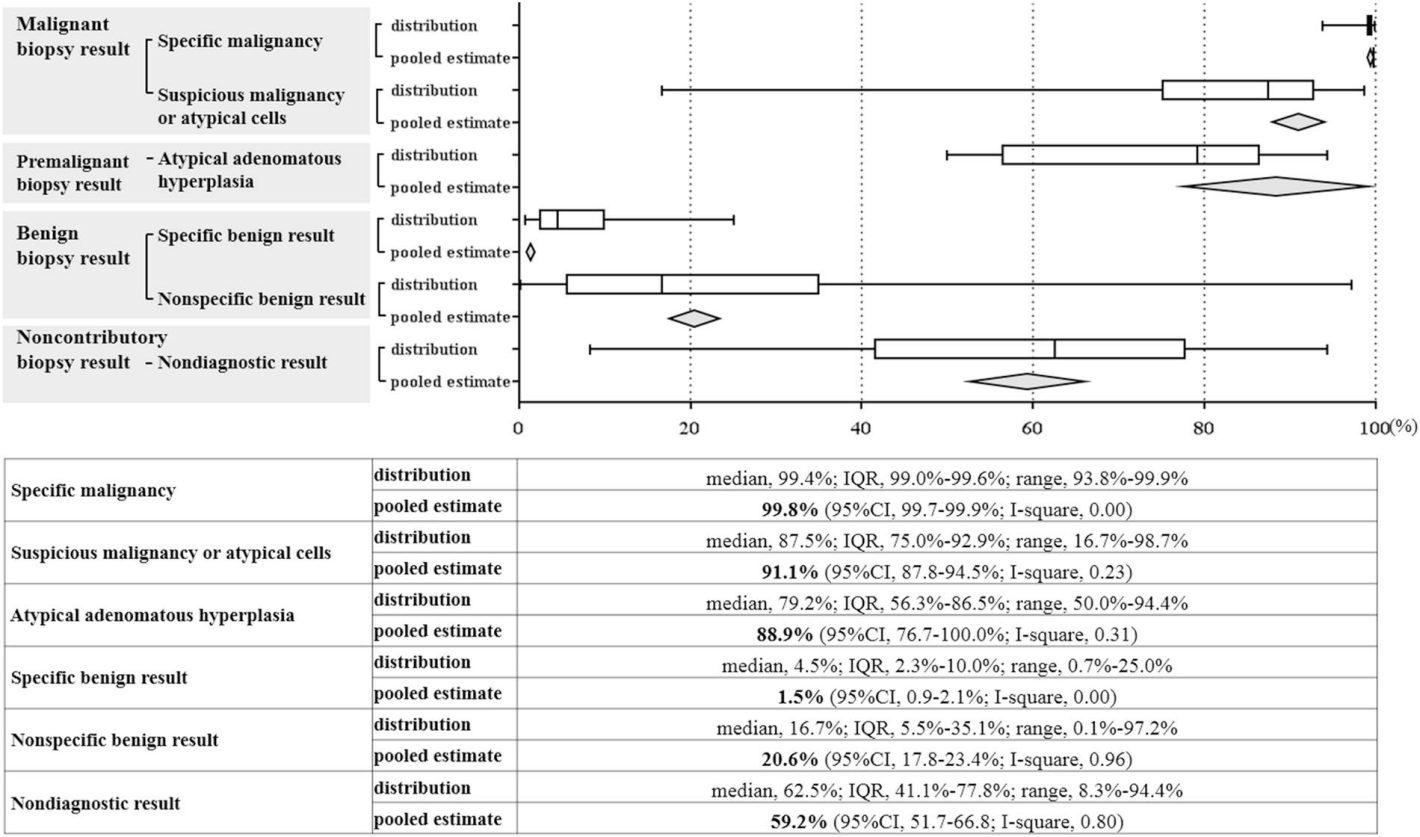
Malignancy rate according to pathology reports of biopsy specimen in percutaneous thoracic needle biopsy.

The final malignancy rate differed according to the PTNB pathology findings (Fig. 3). The pooled final malignancy rate of non-diagnostic results was 59.3% (95% CI, 51.7–66.8%; I^2^ = 0.80, 709 biopsies in 47 studies)^4,6–8,11,12,19,22,23,26–28,33,34,38,40,42,45,48,52,54,55,59–61,65,70,72,76,78,79,81–83,85,91,92,96,101,108,115,129,137–140,147^. The malignancy rate was moderately positively correlated with the overall prevalence of malignancy in the study (correlation coefficient, 0.66; 95% CI, 0.42–0.91).

With regard to other pathology findings, the malignancy rate was the highest for specific malignancy (99.8%, 95% CI, 99.7–99.9%; I^2^ = 0.00; 11,884 biopsies in 114 studies)^3,4,6–9,11–23,25–68,70,72–76,78,79,81–86,88,90–93,96,99–101,103–105,108,113–115,118–120,124,126–130,132,134,138–147^, followed by atypical cells (91.1%, 95% CI, 87.8–94.5%; I^2^ = 0.23; 312 biopsies in 41 studies)^4,9,13,14,17–20,26,27,29,30,34,37,41,42,46,47,53,64,66,70,74–76,79,85,86,89–91,98,101,104,108,115,120,126,129,144,147^, AAH (88.9%, 95% CI, 76.7–100.0%; I^2^ = 0.31; 18 biopsies in 6 studies)^8,76,84,126,128,138^, non-specific benign results (20.6%, 95% CI, 17.8–23.4%; I^2^ = 0.96; 2574 biopsies in 64 studies)^4,6,8,11,12,19,22,23,26–28,30,33,34,38,40,42,44,45,47–49,52,59–62,65,67,69,70,72,74–76,78,79,81–86,91,96,101,108,110,115,119,120,124,126,129,130,132,133,137–140,143,146,147^, and specific benign results (1.5%, 95% CI, 0.9–2.1%; I^2^ = 0.00; 1601 biopsies in 84 studies)^4,6,9,12–16,18,21–23,26–28,30,31,33–40,42,43,45,46,48,50–53,56–65,67–70,72–76,79,81–83,85–88,90–93,98–101,103,104,108,110,114,115,118–121,124,126–130,132,134,139–141,143–145,147^.

### Impact of non-diagnostic results on the diagnostic performance of PTNB

**Figure 4:**
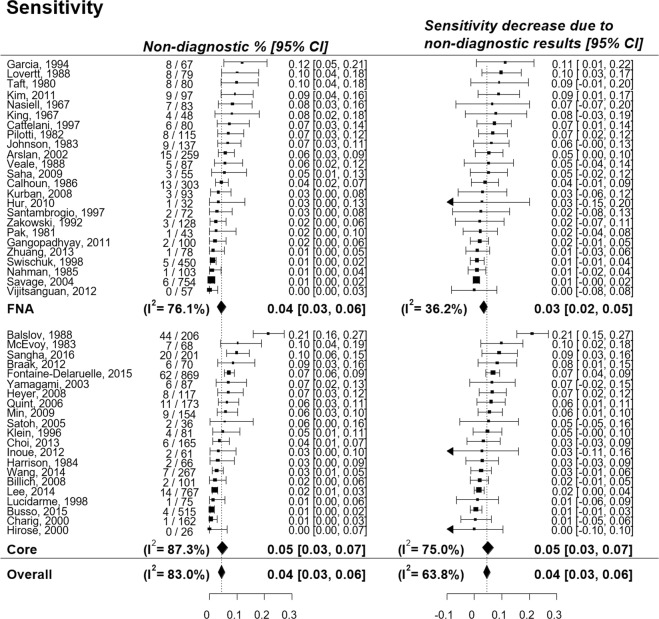
Percentage decrease of sensitivity of percutaneous transthoracic needle biopsy due to non-diagnostic results.

**Figure 5:**
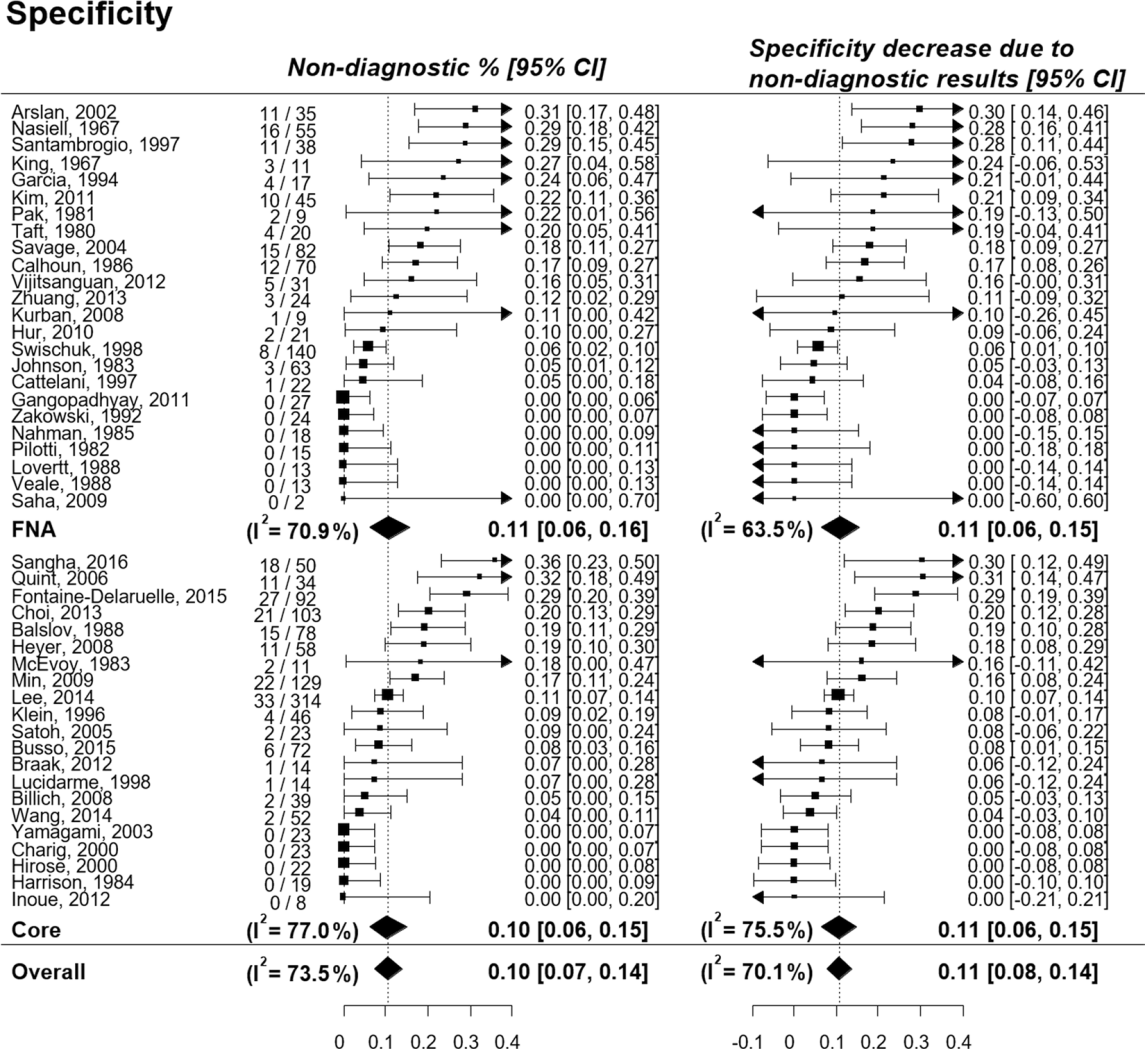
Percentage decrease of specificity of percutaneous transthoracic needle biopsy due to non-diagnostic results.

The pooled percentage decrease of sensitivity and specificity due to non-diagnostic results was 4.5% (95% CI, 3.2–5.7%; I^2^ = 0.64) and 10.7% (95% CI, 7.7–13.7%; I^2^ = 0.70), respectively (Figs 4, 5). The pooled incidence of non-diagnostic results was 4.4% (95% CI, 3.2–5.8%; I^2^ = 0.83) in lesions with a final diagnosis of malignancy and 10.4% (95% CI, 7.5–13.8%; I^2^ = 0.74) in lesions with a final diagnosis of benign disease, resulting in a larger reduction of specificity than of sensitivity. The subgroup analysis according to the biopsy method showed that changes in the pooled sensitivities and specificities were similar between the biopsy methods (Supplementary Fig. A2).

Funnel plot asymmetry was assessed for the incidence of non-diagnostic PTNB results presented in 106 studies. The double arcsine-transformed incidence was used to stabilize variance^149^. The funnel plots were not asymmetrical and the *P* value for the Egger test was 0.291, indicating no obvious publication bias. (Supplementary Fig. A3).

## Discussion

Our meta-analysis revealed that 6.8% of successful PTNB procedures averagely did not offer any information for differentiating malignancies from benign disease. The pooled malignancy rate of non-diagnostic PTNB results was 59.3%, which was much higher than that of non-specific and specific benign results (20.6% and 1.5%, respectively). Although we pooled the incidence and malignancy rates to suggest a summary of those estimates, there was substantial heterogeneity across studies. Non-diagnostic results decreased the sensitivity and specificity of PTNB by 4.5% and 10.7%, respectively, which followed the pooled incidence of non-diagnostic results of 4.4% in lesions ultimately diagnosed as malignancies and those of 10.4% in lesions with a final diagnosis of benign disease. Additionally, multivariate meta-regression analysis revealed that the core biopsy showed significantly less frequent non-diagnostic results than FNA (P = 0.015).

Non-diagnostic PTNB results included specimens with blood, necrosis, normal lung parenchyma, or insufficient tissue to make any diagnosis, and accounted for 6.8% of PTNB specimens. Although the incidence of non-diagnostic results was significantly lower in core biopsy than in FNA, substantial heterogeneity still existed when studies were separately pooled according to the biopsy method. Presumably, lesion characteristics, such as location, distance from the pleura, and necrotic proportions of a lesion affect the likelihood of obtaining a non-diagnostic result^3,91^, although such characteristics could not be considered in this meta-regression analysis.

In non-diagnostic results, the median and pooled estimates of the malignancy rate were 62.5% and 59.3%, respectively, meaning that subsequent diagnostic procedures such as repeated biopsy, surgical exploration, or other imaging investigations such as positron emission tomography were clinically necessary. However, the malignancy rate was substantially heterogeneous across the studies. The heterogeneity seems to have mainly originated from the heterogeneous prevalence of malignancy across studies, given the moderate correlation between the malignancy rate and the overall prevalence of malignancy in the study (correlation coefficient, 0.66). This could result from differences in the study population and the institutional practice of PTNB across the studies.

Non-specific benign results had a relatively high proportion of final malignancy diagnoses (20.6%; 95% CI, 17.8–23.4%) when compared to specific benign results (1.5%; 95% CI, 0.9–2.1%). Similarly to non-diagnostic results, non-specific benign results often required a repeated PTNB or a more invasive procedure such as surgical exploration, especially when there was a discrepancy between a high clinical suspicion of malignancy and non-specific benign PTNB results^150^. Furthermore, lesions with a final diagnosis of malignancy were frequently accompanied by AAH, for which the proportion of final malignancy diagnoses (88.9%; 95% CI, 76.7–100.0%) was close to that of atypical cells or suspicious for malignancy (91.1%; 95% CI, 87.8–94.5%). Although AAH was defined as a peripheral focal proliferation of atypical cuboidal or columnar epithelial cells along the alveoli and respiratory bronchioles^151^, it is a premalignant lesion that forms a continuous spectrum with adenocarcinoma, and a clear distinction between AAH and pre- or minimally-invasive adenocarcinoma cannot be conclusive in PTNB specimens.

By using both the conventional 2-by-2 table approach and the intention-to-diagnose approach, we found that non-diagnostic results decreased the sensitivity and specificity of PTNB by 4.5% and 10.7%, respectively. Interestingly, the degree of reduction in sensitivity and specificity were almost the same as the incidence of non-diagnostic results in lesions with a final diagnosis of malignancy and benign disease, respectively. This is because the occurrence of non-diagnostic results directly led to the decrease of diagnostic accuracy as the accuracy of PTNB in a 2-by-2 table analysis was close to 100% (Figs 4, 5 and Supplementary Table A3).

Our study has several limitations. First, the degree of suspicion (pretest probability) for malignancy could not be considered in our analysis. Second, we regarded repeat PTNBs as separate initial PTNBs, and inter-exam correlations between repeat PTNBs could not be considered due to difficulties in accessing the raw data. Third, the reasons for statistical heterogeneity were not fully identified despite the meta-regression analysis. A detailed examination of the lesion characteristics could have helped identify the causes of heterogeneity, but this information was not extractable from the included studies. Additionally, meta-regression analysis regarding the experience and subspecialty of operators was not included due to the limited number of included studies. Fourth, some inconsistencies existed across the studies in terms of the reference standard for the final diagnosis of malignancy, such as different durations of post-PTNB observation or different rates of surgical confirmation, even though most studies included in our analysis were similar. Fifth, we included studies consisting of 50 or more PTNBs of focal parenchymal lung lesions by referring to the American College of Chest Physicians (ACCP) guideline for the diagnosis of lung cancer in 2013^2^. Although this might potentially cause a biased inclusion of relevant studies, most of the studies (15/21) applied a particular inclusion criteria including pulmonary lesions difficult to be biopsied (9/21, 42.9%), cancer or benign lesion only (4/21, 19.0%), or a new biopsy technique (2/21, 9.5%). Other 6 (28.6%) studies were ineligible to construct a 2 × 3 table. Lastly, we did not include ultrasonography-guided biopsy, because we tried to investigate the diagnostic performances of intrapulmonary lesion, and it was not able to separate the diagnostic performance for subpleural lesions from that for chest wall lesion in the ultrasonography-guided biopsy studies.

In conclusion, the pooled incidence of non-diagnostic results was 6.8% in PTNB procedures and more than half of the non-diagnostic results were from malignancies. In in the 3-by-2 table approach, non-diagnostic results averagely decreased specificity and sensitivity by 10.7% and 4.5%, respectively, which followed incidences of non-diagnostic results in benign disease and malignancies. Because the previous 2 × 2 table analysis could result in reporting a biased diagnostic accuracy, accurate information of non-diagnostic rate can be transmitted to the patients more accurately with the approach of the 3 × 2 table analysis. Additionally, as true malignancy might be masked averagely in 60% of non-diagnostic biopsy results, thoracic interventionists should continue their efforts to minimize non-diagnostic results to maintain the diagnostic accuracy of PTNB, along with discreetly making an effort to identify the actual pathology of pulmonary lesions with the non-diagnostic results.

## Methods

### Search strategy

Two authors (K.J.C and S.H.Y) independently performed literature searches of the Ovid-MEDLINE and Embase databases to identify relevant publications using keywords related to ‘lung,’ ‘biopsy,’ and ‘accuracy’ (Supplementary Table A4), and the searches of two authors were harmonized by consensus. Searches were limited to English-language publications and human studies published through March 2016.

### Inclusion criteria

The following inclusion criteria were applied to determine eligibility: (i) study population consisting of 50 or more PTNBs of focal parenchymal lung lesions^2^; (ii) study fully or partly addressing the diagnostic performance of PTNB; (iii) radiological guidance of fluoroscopy, CT, cone-beam CT, or CT fluoroscopy; (iv) a sufficient description of the data for outcomes to be extracted. In cases of partially or completely overlapping study populations, the study with the most biopsies was included. Case reports, review articles, editorials, letters, comments, and conference proceedings were excluded.

### Definition of outcomes

We assessed 3 outcomes related to non-diagnostic results in this meta-analysis: (1) the incidence of non-diagnostic results, along with that of other pathology findings of PTNB specimens; (2) the final malignancy rate of non-diagnostic results, along with that of PTNB specimens with other pathology findings; (3) the impact of non-diagnostic results on the diagnostic performance of PTNB.

The pathology findings of PTNB specimens were divided into 6 categories which consisted of malignant, premalignant, benign, non-diagnostic findings: specific malignancy, atypical cells or suspicious for malignancy, atypical adenomatous hyperplasia (AAH), non-diagnostic results, non-specific benign disease, and specific benign diseases^84,91^. Specific benign diseases included benign lung tumors, infectious pneumonia, pulmonary tuberculosis, silicosis, vasculitis, or others. Non-specific benign disease referred to acute or chronic non-specific inflammation, granuloma, focal fibrosis, or a specimen without evidence of malignancy^150^. Non-diagnostic results were defined as a pathologic report of PTNB specimen only having blood, necrosis, normal lung parenchyma, or insufficient tissue to make any diagnosis. The final diagnosis was confirmed by the pathologic evaluation of a surgical specimen or clinico-radiologic follow-up for 1 year or longer^2^. Repeated biopsies for the same lesion were regarded as separate initial PTNBs.

To evaluate the impact of non-diagnostic results on the diagnostic performance of PTNB, we constructed a 3-by-2 table where a non-diagnostic PTNB results were added to the middle of the rows between positive and negative PTNB results on a per-biopsy basis. Positive PTNB results included specific malignancy and atypical cells or suspicious for malignancy, whereas negative PTNB results included AAH, non-specific benign disease, and specific benign diseases.

### Data extraction and quality assessment

Data extraction and quality assessment were performed independently by 2 authors (K.J.C and S.H.Y). The quality of the included studies was assessed based on the QUADAS-2 criteria^152^. In case of disagreement between the two authors, a consensus was reached through further discussion with rechecking the text of the study.

### Statistical analysis

A random-effects model was used to estimate the pooled incidences of pathology findings in PTNB specimens and the pooled proportion of final malignancy diagnoses according to the pathology findings of PTNB. The pooled estimates were shown with the distribution of individual study results instead of funnel plot as the funnel plot could not be presented in the text due to large numbers of studies included in the meta-analyses. Statistical heterogeneity across the included studies was assessed using forest plots and the I-squared statistic. To explore reasons for between-study heterogeneity, meta-regression was performed for the incidences of pathology finding in PTNB specimens. The correlation between the final malignancy rate of the non-diagnostic results and the prevalence of malignancy was estimated using a bivariate generalized linear model^153^.

To evaluate the diagnostic accuracy of PTNB, 2 approaches of handling non-diagnostic results were applied: the conventional approach of excluding non-diagnostic results and a conservative intention-to-diagnose approach^10^ (Tables 1 and 2). In the intention-to-diagnose approach, sensitivity and specificity were calculated as the proportion of positive PTNB results among technically successful procedures with a final malignancy diagnosis and the proportion of negative PTNB results among technically successful procedures with a final diagnosis of benign disease, respectively.

**Table 1:** Definition of sensitivity and specificity in the conventional approach.

		Biopsy result	
		Malignancy	Benign
Final result	Malignancy	a (true positive)	c (false negative)
	Benign	b (false positive)	d (true negative)

$$\begin{array}{c}{\bf{Sensitivity}}{\boldsymbol{:}}\\ \frac{{\rm{The}}\,{\rm{number}}\,{\rm{of}}\,{\rm{procedures}}\,{\rm{with}}\,{\rm{final}}\,{\rm{malignancy}}\,{\rm{and}}\,{\rm{a}}\,{\rm{positive}}\,{\rm{biopsy}}\,{\rm{result}}\,({\rm{a}})}{\,\,{\rm{The}}\,{\rm{number}}\,{\rm{of}}\,{\rm{procedures}}\,{\rm{having}}\,{\rm{a}}\,{\rm{diagnostic}}\,{\rm{result}}\,\& \,{final}\,{malignancy}\,(a+c)}.\end{array}$$

$$\begin{array}{c}{\bf{Specificity}}{\boldsymbol{:}}\\ \frac{{\rm{The}}\,{\rm{number}}\,{\rm{of}}\,{\rm{procedures}}\,{\rm{with}}\,{\rm{final}}\,{\rm{benign}}\,{\rm{result}}\,{\rm{and}}\,{\rm{a}}\,{\rm{negative}}\,{\rm{biopsy}}\,{\rm{result}}\,({\rm{d}})}{\,\,{\rm{The}}\,{\rm{number}}\,{\rm{of}}\,{\rm{procedures}}\,{\rm{having}}\,{\rm{a}}\,{\rm{diagnostic}}\,{\rm{result}}\,\& \,{final}\,{benign}\,{\rm{result}}\,({\rm{b}}+{\rm{d}})}.\end{array}$$

**Table 2:** Definition of sensitivity and specificity in the intention-to-diagnose approach.

		Biopsy result		
		Malignancy	Non-diagnostic result	Benign
Final result	Malignancy	a (true positive)	e	c (false negative)
	Benign	b (false positive)	f	d (true negative)

$$\begin{array}{c}{\bf{Sensitivity}}{\boldsymbol{:}}\\ \frac{{\rm{The}}\,{\rm{number}}\,{\rm{of}}\,{\rm{procedures}}\,{\rm{with}}\,{\rm{final}}\,{\rm{malignancy}}\,{\rm{and}}\,{\rm{a}}\,{\rm{positive}}\,{\rm{biopsy}}\,{\rm{result}}\,({\rm{a}})}{\,\,\,{\rm{The}}\,{\rm{number}}\,{\rm{of}}\,{\rm{technically}}\,{\rm{succeeded}}\,{\rm{procedures}}\,{\rm{with}}\,{final}\,{malignancy}\,({\rm{a}}+{\rm{e}}+{\rm{c}})}.\end{array}$$

$$\begin{array}{c}{\bf{Specificity}}{\boldsymbol{:}}\\ \frac{{\rm{The}}\,{\rm{number}}\,{\rm{of}}\,{\rm{procedures}}\,{\rm{with}}\,{\rm{final}}\,{\rm{benign}}\,{\rm{result}}\,{\rm{and}}\,{\rm{a}}\,{\rm{negative}}\,{\rm{biopsy}}\,{\rm{result}}\,({\rm{d}})}{\,\,{\rm{The}}\,{\rm{number}}\,{\rm{of}}\,{\rm{technically}}\,{\rm{succeeded}}\,{\rm{procedures}}\,{\rm{with}}\,{final}\,{benign}\,{\rm{result}}\,({\rm{b}}+{\rm{f}}+{\rm{d}})}.\end{array}$$

A trivariate generalized linear model^153^ was used to explore correlations of the 2 diagnostic measures of sensitivity and specificity with prevalence, as the diagnostic measures may vary with prevalence due to different definitions of the reference standard or different distributions of disease severity^154^. Since negligible correlations between prevalence and the 2 diagnostic measures were observed in the trivariate generalized linear model, a bivariate generalized linear model^155^ was employed to estimate the pooled percentage decrease of sensitivity and specificity due to non-diagnostic results. The incidence of non-diagnostic results was examined by final disease status in order to explore the reason for different degrees of decrease between sensitivity and specificity in the intention-to-diagnose analysis. Subgroup analysis was conducted for the biopsy method (core biopsy versus FNA), as the biopsy method may affect the diagnostic accuracy.

The potential for publication bias was visually evaluated using funnel plots and the Egger test for asymmetry^156^. Analyses were performed using MetaAnalyst version 3.1 (Tufts Medical Center, Boston, MA, USA)^157^, the NLMIXED procedure in SAS 9.3 (SAS Corp., Cary, NC, USA), and the *metafor* package^158^ in R 3.4.0.

## Supplementary information

Supplementary information

## Data Availability

The datasets generated during and/or analyzed during the current study are available from the corresponding author on reasonable request.
