# Supplementary material for: Non-diagnostic Results of Percutaneous Transthoracic Needle Biopsy: A Meta-analysis
Source: Sci Rep. 2019 Aug 27;9:12428. doi: 10.1038/s41598-019-48805-x (PMC6711972; doi:10.1038/s41598-019-48805-x)

SUPPLEMENTARY INFORMATION

TITLE: Non-diagnostic Results of Percutaneous Transthoracic Needle Biopsy: A Meta-analysis

Authors' full names: Kum Ju Chae[1], Hyunsook Hong[2], Soon Ho Yoon[3], Seokyung Hahn[4], Gong Yong Jin[1], Chang Min Park[3], Jin Mo Goo[3,5]

Authors' affiliation(s): [1]Department of Radiology, Institute of Medical Science, Research Institute of Clinical Medicine of Chonbuk National University-Biomedical Research Institute of Chonbuk National University Hospital; [2]Medical Research Collaborating Center, Seoul National University Hospital, Seoul, Korea; [3]Department of Radiology, Seoul National University College of Medicine, Seoul National University Hospital, Seoul, Korea; [4]Department of Medicine, Seoul National University College of Medicine, Seoul, Korea; [5]Institute of Radiation Medicine, Seoul National University Medical Research Center, Seoul, Korea

Supplemental Tables

Supplemental Table A1. Summary of characteristics of the included studies

Source	Total biopsy No.	Lesion size (mm)[a]	Biopsy Needle[b]	Needle Guide[c]	Prevalence of malig.	Biopsy result						FU loss
						Technically succeed biopsies						
						Malignant PTNB result		Benign PTNB result			Non-diag.	
						Specific malignancy	Susp.	AAH	Specific benign	Non-specific benign.		
King, et al. 1967	59	range, 15-150	FNA	F	81.4%	42/0	0		0	2/8	4/3	0
Nasiell, et al. 1967	144	unknown	FNA	F	60.1%	60/0	0		0	16/39	7/16	6
Stevens, et al. 1968	100	unknown	FNA	F	62.0%	52/2	4/0		0/20	6/16		0
Pavy, et al. 1974	59	range,20-85	FNA	F	91.1%	40/0	3/0		0	8/5		3
Francis, et al. 1977	244	range, 5-150	FNA	F	67.6%	135/4	0		0/12	30/63		0
House, et al. 1977	88	mainly, 20-40	FNA	F	64.7%	53/1	0		0/11	2/18		3
Lalli, et al. 1978	1223	unknown	FNA	F	78.5%	801/1	0/2	144/256				19
Flower, et al. 1979	300	range, 10-100	FNA	F	71.3%	162/3	15/3		0/18	24/57		18
Poe, et al. 1980	103	range, 5-100	FNA	F	77.7%	63/1	9/0		0/2	8/20		0
Taft, et al. 1980	100	unknown	FNA	F	80.0%	60/0	6/1		6/15		8/4	0
Westcott, et al. 1980	403	range, >6	FNA	F	73.3%	284/4	4/2	5/101				3
Allison, et al. 1981	160	unknown	FNA	F	62.7%	85/0	0		0/12	9/44		10

Pak, et al. 1981	52	range, 10-110	FNA	F	82.7%	42/0	0		0/5	0/2	1/2	0
Pilotti, et al. 1982	130	range, 15-80	FNA	F	88.5%	106/1	0		0/5	1/9	8/0	0
Samuelsson, et al. 1982	412	median, >20	FNA	F	N/A	*	*	*	*	*	29†	*
Vine, et al. 1982	100	unknown	FNA	F	N/A	55/0	0		11†	25†		9
Johnson, et al. 1983	200	unknown	FNA	F	68.5%	120/1	2/1		0/31	6/27	9/3	0
McEvoy, et al. 1983	81	unknown	CB	F	86.1%	59/0	2/0		0/4	0/5	7/2	2
Harrison, et al. 1984	89	range,<br>20-80 (89%)	CB	F	77.6%	63/0	0		0/6	1/13	2/0	4
Stevens, et al. 1984	348	unknown	FNA	F	64.1%	192/0	13/1		18/124			0
Crosby, et al. 1985	180	median,30-60	FNA	F	N/A	109/0	7/0		0	35†	29†	0
Greene, et al. 1985	150	mean, 32-37<br>(range, 8-120)	FNA	F	N/A	114/0	0		0/10	4/17		5
Lees, et al. 1985	86	unknown	FNA	F	82.7%	57/0	0	10/14				5
Nahman, et al. 1985	125	unknown	FNA	F	85.1%	101/1	0		0	1/17	1/0	4
Calhoun, et al. 1986	397	unknown	FNA	F	81.2%	252/0	13/0		0/10	25/48	13/12	24
Winning, et al. 1986	181	mean, 39-46	FNA	F	76.4%	97/0	0		0/4	29/35		16
Stanley, et al. 1987	447	unknown	FNA	F, CT	73.4%	312/4	0		0/44	11/69		7
Weisbrod, et al. 1987	133	unknown	FNA	F	71.4%	69/0	1/0		0/3	20/33		7
Balslov, et al. 1988	284	unknown	CB	F	72.5%	161/0	0		0/53	1/10	44/15	0
Levine, et al. 1988	59	unknown	FNA	F	57.6%	24/0	0		0/2	10/23		0
Lovett, et al. 1988	92	mean, 27<br>(15-90)	FNA	F	85.9%	71/0	0		0/4	0/9	8/0	0

Simpson, et al. 1988	233	unknown	FNA	F	93.5%	164/0	7/0	30/14				18
Veale, et al. 1988	100	unknown	FNA	F	87.0%	73/0	3/0		0/8	6/5	5/0	0
Collins, et al. 1992	129	unknown	FNA	F	91.5%	111/0	0		0/4	7/7		0
Cristallini, et al. 1992	390	unknown	FNA	F, CT	N/A	221/1	0		13/67		17†	71
Zakowski, et al. 1992	164	unknown	FNA	F, CT	84.2%	107/0	0		0/3	18/21	3/0	12
Grode, et al. 1993	307	unknown	FNA	F	77.9%	141/0	16/0		0	82/68		0
Burbank, et al. 1994	60	unknown	CB	CT	75.0%	42/0	1/0		1/14	1/1	0	0
Garcia, et al. 1994	84	mean, 29±9a	FNA	CT	79.8%	56/0	0		0/5	3/8	8/4	0
Bocking, et al. 1995	340	unknown	FNA	CT	N/A	250/3	0		*	3/59	*	25
Gasparini, et al. 1995	652	mean, 35 (range, 8-80)	FNA	F	77.3%	425/1	0		0/61	31/72		62
Milman, et al. 1995	103	unknown	FNA	F	75.7%	54/0	0		0	24/25		0
Klein, et al. 1996	133	mean, 29±22	CB	F, CT	63.8%	77/0	0		0/28	0/14	4/4	6
Li, et al. 1996	97	median, >20 (rrange, 4-82)	FNA	CT	87.6%	64/0	13/0		0	8/12		0
Cattelani, et al. 1997	119	median, 20-30	FNA	CT	78.4%	74/0	0		0/21		6/1	17
Santambrogio, et al. 1997	110	range, 10-30	FNA	CT	65.5%	63/1	0		7/26		2/11	0
Westcott, et al. 1997	75	mean, 11 (range, <15)	FNA	F, CT	61.3%	43/0	0		0/5	3/24		0
Yankelevitz, et al. 1997	114	median, 10-20	FNA	CT	74.6%	80/0	0		0/3	5/26		0
Larscheid, et al. 1998	130	median, 30-40	FNA	CT	95.4%	95/0	0		0/2	9/3		21
Lucidarme, et al. 1998	91	mean, 33 (range, 9-80)	CB	CT	84.3%	70/0	0		0/10	4/3	1/1	2

Swischuk, et al. 1998	612	range, 3-100	FNA	F, CT	76.3%	430/2	0		0/55	15/75	5/8	22
Charig, et al. 2000	185	mean, 41.8 (range,13-112)	CB	CT	87.6%	150/0	0		0/16	11/7	1/0	0
Hirose, et al. 2000	48	mean, 23 (range, 8-60)	CB	CTF	54.2%	25/0	0		0/10	1/12	0	0
Laurent, et al. 2000	202	median, >20 (range, 8-140)	CB	CT	79.8%	149/0	0		0/20	9/20		4
Lopez, et al. 2001	79	median, 16-25	CB	CT	63.3%	48/0	1/0		0/8	1/21		0
Arslan, et al. 2002	316	mean, 39±16	FNA	CT	88.1%	228/0	0		0/12	12/16	11/15	22
Wallace, et al. 2002	61	median,8-10 (range, 5-10)	FNA	CT	68.4%	29/0	3/0	7/18				4
Yu, et al. 2002	52	median, 54 (18-150)	CB	CT	91.2%	30/0	0		0/0	1/3	0	18
Anderson, et al. 2003	195	mean, 41 (range, 8-100)	FNA	CT	86.2%	132/0	0		0/13	36/14		0
Geraghty, et al. 2003	856	mean, 30 (range, 4-150)	FNA	CT	N/A	526†	65†		16†	221†	28†	0
Yamagami, et al. 2003	110	mean, 19±9 (range, 3-45)	CB	CTF	79.1%	76/0	1/0		0/8	4/15	6/0	0
Mullan, et al. 2004	53	unknown	FNA	CT	N/A	*	*	*	*	*	9†	*
Savage, et al. 2004	836	mean, 27 (range, 5-100)	FNA	CT	90.2%	741/0	0		0/21	7/46	6/15	0
Gupta, et al. 2005	176	median, 10-20 (range, <20)	FNA	CT	N/A	104/0	0		5/34		21†	12
Loubeyre, et al. 2005	75	median, 30-40	CB	CT	83.6%	61/0	1/0		0/9	0/2	0	2
Mazza, et al. 2005	321	range, 5-80	FNA	CT	N/A	229/2	8/0		0/16	1/19	46†	0
Satoh, et al. 2005	60	mean, 34 (range, 7-120)	CB	CT	75.0%	33/0	0	0/1	0/14	1/7	2/2	0
Bakhshayesh Karam, et al. 2006	505	mean, 56 (range, 20-180)	FNA	CT	N/A	*	*	*	*	*	95†	*

Lourenco, et al. 2006	89	median, >38	FNA	CT	N/A	52/0	3†		4†	0/13	4/2	11
Quint, et al. 2006	226	mean, 36±20<br>(range, 9-160)	CB	CT	83.6%	154/0	3/0		0/7	5/16	11/11	19
Halloush, et al. 2007	132	unknown	FNA	F, CT	N/A	*	*	*	*	*	2†	*
Priola, et al. 2007	612	mean, 36<br>(range, 7-1103	FNA	CT	83.0%	457/1	13†		0/21	16/48	35/21	0
Billich, et al. 2008	130	mean, 29	CB	CT	77.7%	99/0	0		0/4	0/23	2/2	0
Heyer, et al. 2008	175	mean, 46±30<br>(range, 9-157)	CB	CT	66.9%	109/0	0		0/28	0/19	8/11	0
Kim, et al. 2008	53	mean, 19±9<br>(range, 7-45)	CB	CT	80.4%	33/0	0	1/1	0/8	3/0	0	7
Kurban, et al. 2008	102	mean, 36<br>(range, 10-110)	FNA	F	91.2%	78/1	4/0		1/5	7/2	3/1	0
Laspas, et al. 2008	409	range, 6-100	FNA	CT	N/A	290/0	3/1		0/6	25/44	40†	0
Ng, et al. 2008	55	mean, 9<br>(range, 5-10)	FNA	CT	66.0%	21/0			0/1	10/15		8
Chakrabarti, et al. 2009	131	median, 30-40	CB	CT	83.8%	80/0	0		0/3	29/18		1
Guimaraes, et al. 2009	362	mean, 51±24<br>(range, 9-140)	FNA	CT	N/A	204†	38/0		62†		53†	5
Hiraki, et al. 2009	1105	mean, 23±15<br>(range, 4-114)	CB	CTF	78.6%	741/1		42/212				109
Kothary, et al. 2009	139	mean, 20-30<br>(range, 2-80)	CB	CT	72.6%	77/0	13/5		0/17	8/15		4
Min, et al. 2009	291	mean, 31±19	CB	F, CT	54.4%	133/0	10/6		0/55	2/46	9/22	8
Saha, et al. 2009	57	median, 51-100<br>range, 12-136	FNA	CT	96.5%	52/0	0		0/2	0	3/0	0
Uskul, et al. 2009	164	median, 50<br>(range, 13-110)	FNA	CT	83.5%	126/0	0		0/6	11/21		0
Yildirim, et al. 2009	225	mean, 41±20	CB	CT	N/A	*	*	*	*	*	12†	*

Davoudi, et al. 2010	102	unknown	FNA	CT	N/A	*	*	*	*	*	9†	*
Hur, et al. 2010	53	mean, 26±10	FNA	CTF	60.4%	27/0	0		4/19		1/2	0
Kakizawa, et al. 2010	91	mean, 26±10	CB	CT	N/A	*	*	*	*	*	1†	*
Lee, et al. 2010	615	mean, 36 (range, 5-136)	CB	F, CT	N/A	336†	5/0		73†	201†		0
Priola, et al. 2010	321	mean, 37±21 (range, 7-103)	FNA	CT	81.6%	227/1	0		0/29	35/29		0
Schoellnast, et al. 2010	182	mean, 30±20 (range, 5-120)	CB	CT	74.7%	132/1	0		0/21	4/24		0
Gangopadhyay, et al. 2011	127	range, 20-60	FNA	CT	78.7%	96/0	2/0		0/15	0/12	2/0	0
Guimaraes, et al. 2011	97	unknown	CB	CT	N/A	60†	0		8†	15†	3†	11
Kim, et al. 2011	142	mean, 21 (range, 8-50)	FNA	CTF, CT	68.3%	85/0	0		3/35		9/10	0
Lee, et al. 2011	361	mean, 29±9 (range, 5-90)	FNA	CT	66.8%	221/1	0		0/67	16/50		6
Lima, et al. 2011	89	unknown	FNA	CT	79.3%	51/3	10/0		0/3	4/11		7
Matsui, et al. 2011	394	mean, 28±16 (range, 7-115)	CB	CTF	79.9%	262/0	17/70					45
Yamauchi, et al. 2011	52	median, 8-10 (range, <10)	CB	CTF	N/A	*	*	*	*	*	2†	*
Beslic, et al. 2012	242	mean, 29 (range, 12-63)	FNA	CT	N/A	*	*	*	*	*	33†	*
Braak, et al. 2012	84	mean, 32 (range, 3-93)	CB	CBCT	83.3%	63/0	1/0		0/4	0/9	6/1	0
Inoue, et al. 2012	82	mean, 12±4 (range, 5-35)	CB	CTF	88.4%	50/0	0/0	8/0	0/8	1/0	2/0	13
Maataoui, et al. 2012	135	median, 20-30	CB	CT	N/A	*	*	*	*	*	6†	*
McSweeney, et al. 2012	75	mean, 31 (range, 7-107)	CB	CT	N/A	48†	0		10†	13†	4†	0

Nakatani, et al. 2012	107	mean, 30<br>(range, 5-107)	CB	CT	N/A	*	*	*	*	*	2†	*
O'Neill, et al. 2012	164	mean, 35-37	CB	CTF	N/A	*	*	*	*	*	6†	*
Prosch, et al. 2012	326	mean, 26-28<br>(range, 5-124)	CB	CT, CTF	78.5%	192/2	9/53					70
Uruga, et al. 2012	161	unknown	FNA	CT	67.1%	96/0	0		0/24	12/29		0
Vijitsanguan, et al. 2012	94	mean, 46±22<br>(range, 9-110)	FNA	CT	64.8%	54/0	0/1		0/9	3/16	0/5	6
Yoshimatsu, et al. 2012	116	mean, 17-21<br>(range, 4-80)	CB	CTF	N/A	*	*	*	*	*	9†	*
Asai, et al. 2013	102	mean, 28<br>(range, 10-85)	CB	CT	N/A	*	*	*	*	*	10†	*
Choi, et al. 2013	305	mean, 9±1<br>(range, 5-10)	CB	CT	61.6%	148/1	0		3/29	8/52	6/21	37
De Filippo, et al. 2013	198	range, 7-30	FNA	CT	N/A	91/6	0		0/4	17†	41†	39
Li, et al. 2013	169	median, 11-15<br>(range, <20)	CB	CTF	67.5%	103/0	0		0/47	11/8	0	0
Loh, et al. 2013	399	mean, 37<br>(range, 6-123)	FNA	CT	N/A	269/0	3/0		0/53	0/45	29†	0
Malone, et al. 2013	242	median, 18-19	CB	CT	N/A	148†	11†		53†	30†		0
Min, et al. 2013	440	mean, 37-40	CB	CT	N/A	*	*	*	*	*	22†	*
Mondal, et al. 2013	130	unknown	FNA	CT	N/A	*	*	*	*	*	6†	*
Poulou, et al. 2013	994	mean, 37±24	FNA	CT	N/A	506†	0		29†	425†	34†	0
Sconfienza, et al. 2013	170	mean, 24±8	CB	CT	N/A	*	*	*	*	*	6†	*
Tachibana, et al. 2013	270	mean, 31-32<br>(range, 8-11)	CB	CT	77.8%	184/1	5/0	1/1	0/14	20/44	0	0
Tuna, et al. 2013	105	unknown	CB	CT	91.8%	82/0	0		0/5	7/3		8

Yamagami, et al. 2013	85	mean, 14±6 (range, 4-30)	CB	CTF	N/A	58/0	0	2/0	8†	13†	4†	0
Zhuang, et al. 2013	102	mean, 36 (range, 10-70)	FNA	CT	78.4%	72/0	4/2		0/9	1/10	1/3	0
Floridi, et al. 2014	100	mean, 51 (range, 7-140)	CB	CBCT	N/A	68/0	0		0/11	7/9	5†	0
Guimaraes, et al. 2014	459	mean, 42±24	FNA	CT	N/A	*	*	*	*	*	61†	*
Jiao, et al. 2014	108	mean, 46±29	CB	CBCT	84.3%	88/0	0		1/3	2/14	0	0
Konjengbam, et al. 2014	61	unknown	FNA	CT	N/A	38†	2†		4†	12†	5†	0
Kravtsov, et al. 2014	245	unknown	FNA	CT	85.3%	190/0	0		0/1	19/35		0
Lee, et al. 2014	1148	mean, 27±17 (range, 5-130)	CB	CBCT	71.0%	720/0	16/0		1/107	16/174	14/33	67
Mendiratta-Lala, et al. 2014	169	mean, 24 (range, 9-67)	CB	CTF	N/A	*	*	*	*	*	25†	*
Patel, et al. 2014	174	mean, 27±21 (range, 3-114)	CB	CT	N/A	*	*	*	*	*	27†	*
Shrestha, et al. 2014	252	unknown	FNA	CT	N/A	210†	0		19†	11†	9/3	0
Wang, et al. 2014	345	mean, 41 (range, 8-134)	CB	CT	83.7%	250/0	0	3/0	7/50		7/2	26
Busso, et al. 2015	824	mean, 36 (range, 6-150)	CB	CT	87.7%	497/0	0		0/16	14/50	4/6	237
Fontaine-Delaruelle, et al. 2015	980	median, 30	CB	CT	90.4%	776/1	0		7/42	24/22	62/27	19
Jaconi, et al. 2015	375	mean, 39±25 (range, 7-190)	CB	CBCT	N/A	283/0	0		0/46	18†	28†	(3)‡
Schulze, et al. 2015	571	median, 20-30 (range, 4-100)	CB	CT	67.6%	365/0	14/182					10
Takeshita, et al. 2015	750	mean, 24±15	FNA	CT	N/A	541/1	0		0/51	51/96	10†	0
Yaffe, et al. 2015	181	mean, 24±19	CB	CT	92.5%	146/1	5/0		0/9	9/3		8

Haas, et al. 2016	660	mean, 31 (IQR, 17-40)	FNA	CT	81.7%	471/0	0		0/50	42/65		32
Rotolo, et al. 2016	324	mean, 19 (range, 4-30)	CB	CBCT, CTF	N/A	189/0	0		0/76	17/0	28[†]	14
Sangha, et al. 2016	251	mean, 30-32 (range, 7-104)	CB	CT	80.1%	141/0	26/5		0/10	14/17	20/18	0

Abbreviations: AAH = atypical adenomatous hyperplasia; N/A = not available; FU = follow-up; PTNB = percutaneous transthoracic needle biopsy; [a]Size indicates a mean or median size; [b]CB = core biopsy; FNA = fine needle aspiration; [c]F = fluoroscopy; CBCT= cone beam CT; CTF = CT fluoroscopy.Data in cells indicates the number of biopsy. The fractional representation means final malignancy/final benign result.

*Data cannot be extracted.

†Data cannot be separated into final benign or malignancy.

‡Among the 18 non-specific and 28 non-diagnostic results, 3 did not have final result.

Supplemental Table A2. Meta-regression analysis for the incidence of pathologic reports of PTNB results

	Group 1			Group 2			Group 3			Univariate P value	Multivariate P value
	**Incidence (%)**	**I^2**	**Included studies**	**Incidence (%)**	**I^2**	**Included studies**	**Incidence (%)**	**I^2**	**Included studies**		
**Non-diagnostic results**											
Biopsy needle *(FNA vs CB)*	8.0	0.90	58	5.5	0.90	48				<u>*0.010*</u>	<u>*0.015*</u>
Guiding method *(Fluoroscopy vs CT/CBCT vs CTF)*	8.3	0.80	22	5.5	0.92	72	6.5	0.66	12	<u>*0.041*</u>	0.412
Lesion size *(<20mm vs ≥20mm)*	9.0	0.87	10	6.6	0.91	96				0.261	0.087
Publication year *(<2000 vs ≥2000)*	7.7	0.87	31	6.5	0.91	75				0.308	0.547
**Specific malignancy**											
Biopsy needle *(FNA vs CB)*	67.5	0.93	75	69.7	0.92	47				0.285	0.659
Guiding method *(Fluoroscopy vs CT/CBCT vs CTF)*	66.3	0.91	37	70.5	0.92	74	66.8	0.81	11	0.114	0.282
Lesion size *(<20mm vs ≥20mm)*	62.8	0.46	13	69.0	0.93	109				0.057	<u>*0.015*</u>

Publication year *(<2000 vs ≥2000)*	66.9	0.89	50	69.4	0.94	72				0.178	0.903
**Atypia or Suspicious for malignancy**											
Biopsy needle *(FNA vs CB)*	3.5	0.88	29	2.9	0.79	16				0.570	0.430
Guiding method *(Fluoroscopy vs CT/CBCT vs CTF)*	2.9	0.79	17	2.9	0.88	27	7.6	-	1	0.626	0.492
Lesion size *(<20mm vs ≥20mm)*	3.1	0.68	3	3.2	0.87	42				0.816	0.899
Publication year *(<2000 vs ≥2000)*	3.4	0.85	18	3.3	0.86	27				0.964	0.568
**Atypical adenomatous hyperplasia**											
Lesion size *(<20mm vs ≥20mm)*	5.2	0.59	3	0.9	0.00	3				*0.032*	N/A
**Specific benign results**											
Biopsy needle *(FNA vs CB)*	5.1	0.91	57	9.8	0.89	37				*<0.001*	*0.001*

Guiding method *(Fluoroscopy vs CT/CBCT vs CTF)*	3.8	0.93	29	6.4	0.92	61	14.7	0.80	4	*0.006*	0.443
Lesion size *(<20mm vs ≥20mm)*	10.0	0.91	10	6.5	0.92	84				0.129	0.132
Publication year *(<2000 vs ≥2000)*	5.2	0.91	37	7.9	0.92	57				*0.040*	0.689
**Nonspecific benign results**											
Biopsy needle *(FNA vs CB)*	16.2	0.96	35	11.9	0.90	32				*0.039*	*0.035*
Guiding method *(Fluoroscopy vs CT/CBCT vs CTF)*	12.2	0.87	16	13.2	0.92	44	13.3	0.92	7	0.926	0.375
Lesion size *(<20mm vs ≥20mm)*	11.0	0.91	7	14.5	0.94	60				0.290	0.491
Publication year *(<2000 vs ≥2000)*	14.2	0.90	23	14.1	0.95	44				0.942	0.918

Definition of abbreviations: PTNB = percutaneous transthoracic needle biopsy; FNA = fine needle aspiration, CB = core biopsy, N/A = not available. P values underlined in italic type indicate statistical significance.

Supplemental Table A3. Sensitivity and specificity in 2-by-2, 3-by-2 table analyses, and incidence of non-diagnostic results in malignancy and benign disease

Sensitivity in 3 by 2 table:

$$\frac{\text{The number of procedures with final malignancy and a positive biopsy result (a)}}{\text{The number of technically succeeded procedures with final malignancy (a + e + c)}}$$

$$= \frac{\frac{a}{(a+c)}}{\frac{(\mathrm{a+e+c})}{(\mathrm{a+c})}} = \frac{Sensitivity\ in\ 2\ by\ 2\ table}{\frac{(\mathrm{a+e+c})}{(\mathrm{a+c})}} = \frac{Sensitivity\ in\ 2\ by\ 2\ table * (a+c)}{(\mathrm{a+e+c})}$$

$$= Sensitivity\ in\ 2\ by\ 2\ table * (1 - \frac{e}{\mathrm{a+e+c}})$$

= *Sensitivity in 2 by 2 table * [1-Incidence of non-diagnostic results in malignancy]*

Specificity in 3 by 2 table:

$$\frac{\text{The number of procedures with final benign result and a negative biopsy result (d)}}{\text{The number of technically succeeded procedures with final benign result (b + f + d)}}$$

$$= \frac{\frac{b}{(\mathrm{b+d})}}{\frac{(\mathrm{b+f+d})}{(\mathrm{b+d})}} = \frac{Specificity\ in\ 2\ by\ 2\ table}{\frac{(\mathrm{b+f+d})}{(\mathrm{b+d})}} = \frac{Specificity\ in\ 2\ by\ 2\ table * (b+d)}{(b+\mathrm{f+d})}$$

$$= Specificity\ in\ 2\ by\ 2\ table * (1 - \frac{\mathrm{f}}{b+\mathrm{f+d}})$$

= *Specificity in 2 by 2 table * [1- Incidence of non-diagnostic results in benign disease*

Supplemental Table A4.

OVID/MEDLINE Search Strategy

1. exp Lung/

2. Lung.mp

3. Pulmonary.mp

4. $thoracic.mp

5. $thorax.mp

6. Consolidation$.mp

7. 1 or 2 or 3 or 4 or 5 or 6

8. exp biopsy/

9. Biops$.mp

10. Aspiration$.mp

11. Sampling$.mp

12. 8 or 9 or 10 or 11

13. exp “Diagnostic accuracy”/

14. Diagnos$.mp

15. Accurac$.mp

16. Specificit$.mp

17. Sensitivit$.mp

18. Complication$.mp

19. Pneumothorax.mp

20. Hemoptysis.mp

21. 13 or 14 or 15 or 16 or 17 or 18 or 19 or 20

22. 7 and 12 and 21

23. limit 22 to (English language and humans)

EMBASE Search Strategy

#1. Lung

#2. Pulmonary

#3. *thoracic

#4. *thorax

#5. Consolidation*

#6. #1 OR #2 OR #3 OR #4 OR #5

#7. Biops*

#8. Aspiration*

#9. Sampling*

#10. #7 OR #8 OR #9

#11. Diagnos*

#12. Accurac*

#13. Specificit*

#14. Sensitivit*

#15. Complication*

#16. Pneumothorax*

#17. Hemoptysis*

#18. #11 OR #12 OR #13 OR #14 OR #15 OR #16 OR #17

#19. #6 AND #10 AND #18

#20. #19 AND [human]/lim AND [English]/lim

Supplemental Figures

Supplemental Figure A1. Quality assessment of included studies

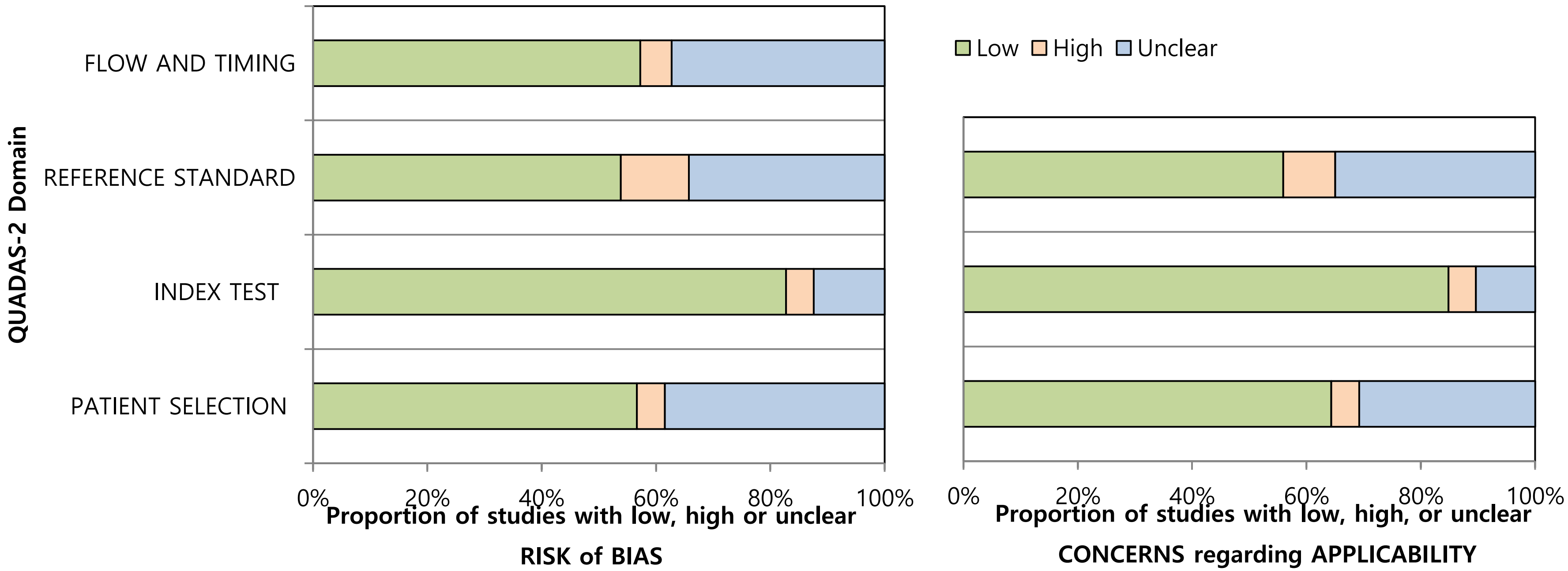

Supplemental Figure A2. Forest plots of prevalence, sensitivity and specificity according to each approach

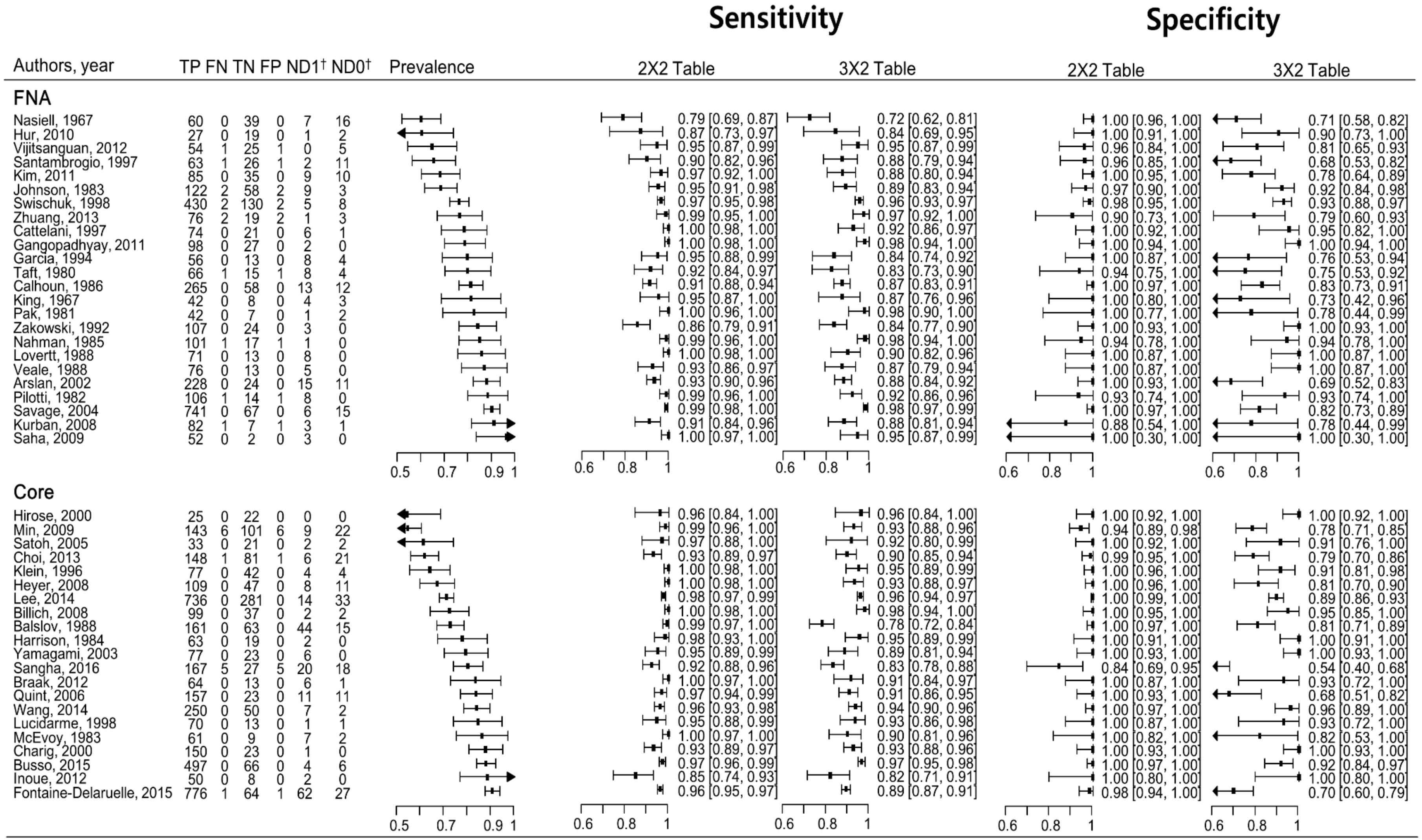

Authors, year	TP	FN	TN	FP	ND1†	ND0†	Sensitivity 2X2 Table	Sensitivity 3X2 Table	Specificity 2X2 Table	Specificity 3X2 Table
**FNA**										
Nasiell, 1967	60	0	39	0	7	16	0.79 [0.69, 0.87]	0.72 [0.62, 0.81]	1.00 [0.96, 1.00]	0.71 [0.58, 0.82]
Hur, 2010	27	0	19	0	1	2	0.87 [0.73, 0.97]	0.84 [0.69, 0.95]	1.00 [0.91, 1.00]	0.90 [0.73, 1.00]
Vijitsanguan, 2012	54	1	25	1	0	5	0.95 [0.87, 0.99]	0.95 [0.87, 0.99]	0.96 [0.84, 1.00]	0.81 [0.65, 0.93]
Santambrogio, 1997	63	1	26	1	2	11	0.90 [0.82, 0.96]	0.88 [0.79, 0.94]	0.96 [0.85, 1.00]	0.68 [0.53, 0.82]
Kim, 2011	85	0	35	0	9	10	0.97 [0.92, 1.00]	0.88 [0.80, 0.94]	1.00 [0.95, 1.00]	0.78 [0.64, 0.89]
Johnson, 1983	122	2	58	2	9	3	0.95 [0.91, 0.98]	0.89 [0.83, 0.94]	0.97 [0.90, 1.00]	0.92 [0.84, 0.98]
Swischuk, 1998	430	2	130	2	5	8	0.97 [0.95, 0.98]	0.96 [0.93, 0.97]	0.98 [0.95, 1.00]	0.93 [0.88, 0.97]
Zhuang, 2013	76	2	19	2	1	3	0.99 [0.95, 1.00]	0.97 [0.92, 1.00]	0.90 [0.73, 1.00]	0.79 [0.60, 0.93]
Cattelani, 1997	74	0	21	0	6	1	1.00 [0.98, 1.00]	0.92 [0.86, 0.97]	1.00 [0.92, 1.00]	0.95 [0.82, 1.00]
Gangopadhyay, 2011	98	0	27	0	2	0	1.00 [0.98, 1.00]	0.98 [0.94, 1.00]	1.00 [0.94, 1.00]	1.00 [0.94, 1.00]
Garcia, 1994	56	0	13	0	8	4	0.95 [0.88, 0.99]	0.84 [0.74, 0.92]	1.00 [0.87, 1.00]	0.76 [0.53, 0.94]
Taft, 1980	66	1	15	1	8	4	0.92 [0.84, 0.97]	0.83 [0.73, 0.90]	0.94 [0.75, 1.00]	0.75 [0.53, 0.92]
Calhoun, 1986	265	0	58	0	13	12	0.91 [0.88, 0.94]	0.87 [0.83, 0.91]	1.00 [0.97, 1.00]	0.83 [0.73, 0.91]
King, 1967	42	0	8	0	4	3	0.95 [0.87, 1.00]	0.87 [0.76, 0.96]	1.00 [0.80, 1.00]	0.73 [0.42, 0.96]
Pak, 1981	42	0	7	0	1	2	1.00 [0.96, 1.00]	0.98 [0.90, 1.00]	1.00 [0.77, 1.00]	0.78 [0.44, 0.99]
Zakowski, 1992	107	0	24	0	3	0	0.86 [0.79, 0.91]	0.84 [0.77, 0.90]	1.00 [0.93, 1.00]	1.00 [0.93, 1.00]
Nahman, 1985	101	1	17	1	1	0	0.99 [0.96, 1.00]	0.98 [0.94, 1.00]	0.94 [0.78, 1.00]	0.94 [0.78, 1.00]
Lovertt, 1988	71	0	13	0	8	0	1.00 [0.98, 1.00]	0.90 [0.82, 0.96]	1.00 [0.87, 1.00]	1.00 [0.87, 1.00]
Veale, 1988	76	0	13	0	5	0	0.93 [0.86, 0.97]	0.87 [0.79, 0.94]	1.00 [0.87, 1.00]	1.00 [0.87, 1.00]
Arslan, 2002	228	0	24	0	15	11	0.93 [0.90, 0.96]	0.88 [0.84, 0.92]	1.00 [0.93, 1.00]	0.69 [0.52, 0.83]
Pilotti, 1982	106	1	14	1	8	0	0.99 [0.96, 1.00]	0.92 [0.86, 0.96]	0.93 [0.74, 1.00]	0.93 [0.74, 1.00]
Savage, 2004	741	0	67	0	6	15	0.99 [0.98, 1.00]	0.98 [0.97, 0.99]	1.00 [0.97, 1.00]	0.82 [0.73, 0.89]
Kurban, 2008	82	1	7	1	3	1	0.91 [0.84, 0.96]	0.88 [0.81, 0.94]	0.88 [0.54, 1.00]	0.78 [0.44, 0.99]
Saha, 2009	52	0	2	0	3	0	1.00 [0.97, 1.00]	0.95 [0.87, 0.99]	1.00 [0.30, 1.00]	1.00 [0.30, 1.00]
**Core**										
Hirose, 2000	25	0	22	0	0	0	0.96 [0.84, 1.00]	0.96 [0.84, 1.00]	1.00 [0.92, 1.00]	1.00 [0.92, 1.00]
Min, 2009	143	6	101	6	9	22	0.99 [0.96, 1.00]	0.93 [0.88, 0.96]	0.94 [0.89, 0.98]	0.78 [0.71, 0.85]
Satoh, 2005	33	0	21	0	2	2	0.97 [0.88, 1.00]	0.92 [0.80, 0.99]	1.00 [0.92, 1.00]	0.91 [0.76, 1.00]
Choi, 2013	148	1	81	1	6	21	0.93 [0.89, 0.97]	0.90 [0.85, 0.94]	0.99 [0.95, 1.00]	0.79 [0.70, 0.86]
Klein, 1996	77	0	42	0	4	4	1.00 [0.98, 1.00]	0.95 [0.89, 0.99]	1.00 [0.96, 1.00]	0.91 [0.81, 0.98]
Heyer, 2008	109	0	47	0	8	11	1.00 [0.98, 1.00]	0.93 [0.88, 0.97]	1.00 [0.96, 1.00]	0.81 [0.70, 0.90]
Lee, 2014	736	0	281	0	14	33	0.98 [0.97, 0.99]	0.96 [0.94, 0.97]	1.00 [0.99, 1.00]	0.89 [0.86, 0.93]
Billich, 2008	99	0	37	0	2	2	1.00 [0.98, 1.00]	0.98 [0.94, 1.00]	1.00 [0.95, 1.00]	0.95 [0.85, 1.00]
Balslov, 1988	161	0	63	0	44	15	0.99 [0.97, 1.00]	0.78 [0.72, 0.84]	1.00 [0.97, 1.00]	0.81 [0.71, 0.89]
Harrison, 1984	63	0	19	0	2	0	0.98 [0.93, 1.00]	0.95 [0.89, 0.99]	1.00 [0.91, 1.00]	1.00 [0.91, 1.00]
Yamagami, 2003	77	0	23	0	6	0	0.95 [0.89, 0.99]	0.89 [0.81, 0.94]	1.00 [0.93, 1.00]	1.00 [0.93, 1.00]
Sangha, 2016	167	5	27	5	20	18	0.92 [0.88, 0.96]	0.83 [0.78, 0.88]	0.84 [0.69, 0.95]	0.54 [0.40, 0.68]
Braak, 2012	64	0	13	0	6	1	1.00 [0.97, 1.00]	0.91 [0.84, 0.97]	1.00 [0.87, 1.00]	0.93 [0.72, 1.00]
Quint, 2006	157	0	23	0	11	11	0.97 [0.94, 0.99]	0.91 [0.86, 0.95]	1.00 [0.93, 1.00]	0.68 [0.51, 0.82]
Wang, 2014	250	0	50	0	7	2	0.96 [0.93, 0.98]	0.94 [0.90, 0.96]	1.00 [0.97, 1.00]	0.96 [0.89, 1.00]
Lucidarme, 1998	70	0	13	0	1	1	0.95 [0.88, 0.99]	0.93 [0.86, 0.98]	1.00 [0.87, 1.00]	0.93 [0.72, 1.00]
McEvoy, 1983	61	0	9	0	7	2	1.00 [0.97, 1.00]	0.90 [0.81, 0.96]	1.00 [0.82, 1.00]	0.82 [0.53, 1.00]
Charig, 2000	150	0	23	0	1	0	0.93 [0.89, 0.97]	0.93 [0.88, 0.96]	1.00 [0.93, 1.00]	1.00 [0.93, 1.00]
Busso, 2015	497	0	66	0	4	6	0.97 [0.96, 0.99]	0.97 [0.95, 0.98]	1.00 [0.97, 1.00]	0.92 [0.84, 0.97]
Inoue, 2012	50	0	8	0	2	0	0.85 [0.74, 0.93]	0.82 [0.71, 0.91]	1.00 [0.80, 1.00]	1.00 [0.80, 1.00]
Fontaine-Delaruelle, 2015	776	1	64	1	62	27	0.96 [0.95, 0.97]	0.89 [0.87, 0.91]	0.98 [0.94, 1.00]	0.70 [0.60, 0.79]

† ND1: # of non-diagnostic results in final malignancy
ND0: # of non-diagnostic results in final benign

Supplemental Figure A3. Funnel plot for the incidence of non-diagnostic PTNB results

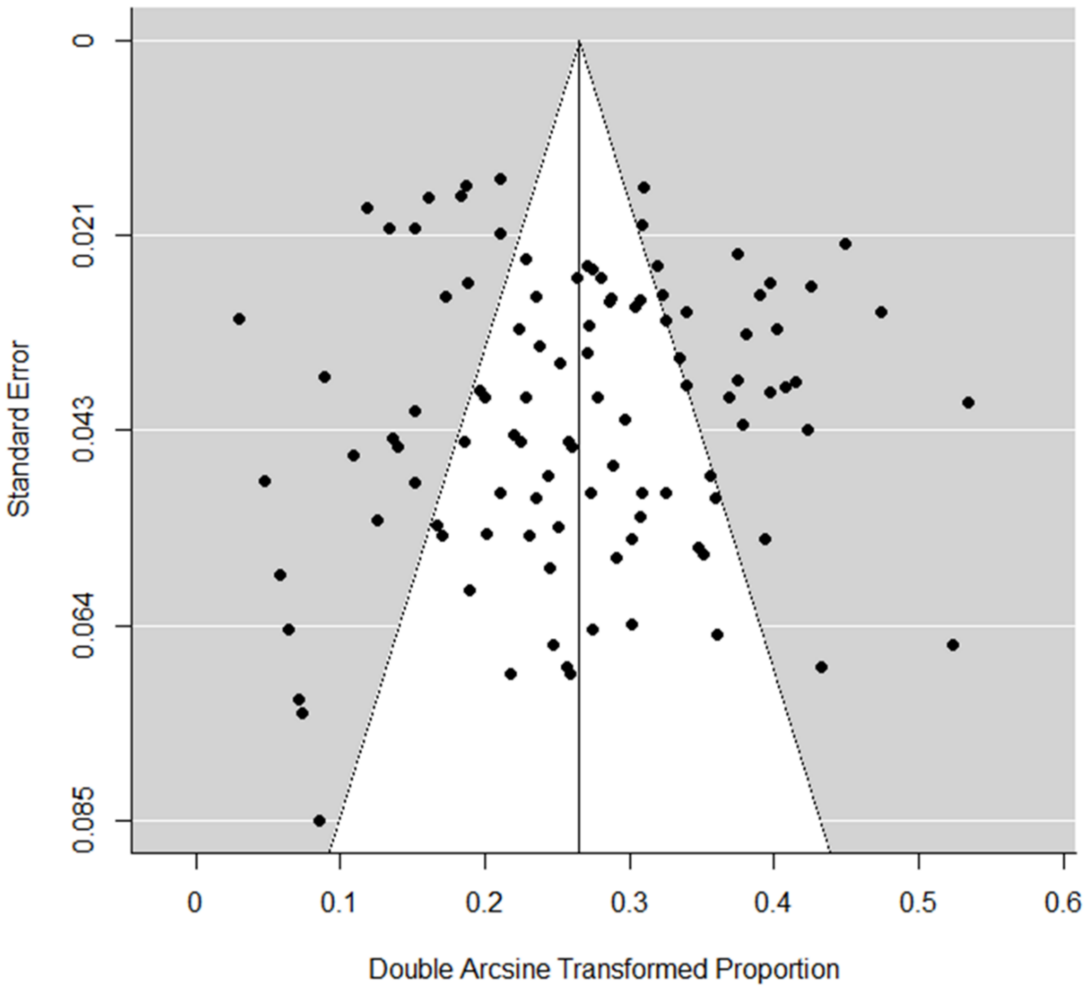

Supplement: Supplementary file 1 — Supplementary information [file 41598_2019_48805_MOESM1_ESM.pdf]
